# A Pyrene@Micelle Sensor for Fluorescent Oxygen Sensing

**DOI:** 10.1155/2015/245031

**Published:** 2015-10-11

**Authors:** Yan-xia Yuan, Hong-shang Peng, Jian-tao Ping, Xiao-hui Wang, Fang-tian You

**Affiliations:** Key Laboratory of Luminescence and Optical Information, Ministry of Education, Institute of Optoelectronic Technology, Beijing Jiaotong University, Beijing 100044, China

## Abstract

For most fluorescent oxygen sensors developed today, their fabrication process is either time-consuming or needs specialized knowledge. In this work, a robust fluorescent oxygen sensor is facilely constructed by dissolving pyrene molecules into CTAB aqueous solution. The as-prepared pyrene@micelle sensors have submicron-sized diameter, and the concentration of utilized pyrene can be reduced as low as 0.8 mM but still can exhibit dominant excimer emission. The excimer fluorescence is sensitive to dissolved oxygen in both intensity and lifetime, and the respective Stern-Volmer plot follows a nonlinear behavior justified by a two-site model. Because of the merits of large Stokes shift (~140 nm), easy fabrication, and robustness, the pyrene@micelle sensors are very attractive for practical determination of oxygen.

## 1. Introduction

Determination of oxygen concentration is important for a number of applications ranging from clinical analysis to environment monitoring [1, 2]. Among the various sensing methods, fluorescence-based techniques have attracted considerable interest because they can be noninvasive and sensitive and work in even strong electromagnetic fields [3, 4]. These fluorescent oxygen sensors usually were constructed by incorporating probe dyes into an inert matrix, taking the form of strip [5], film [6], and nanoparticles [7–9]. Since the probe dyes are trapped by solid matrix, their collision probability with oxygen molecules (e.g., fluorescence quenching) is reduced to some extent, and oxygen sensitivity is undermined accordingly. As an alternative, oxygen sensors based on free probed dyes should have higher sensitivity.

Pyrene is an aromatic, polycyclic hydrocarbon with planer structure, which is widely used as fluorescent chemosensor because of the characteristic photophysical properties such as high quantum yield, long singlet lifetime, and environment-sensitive fluorescence. In terms of oxygen sensing, the long lifetime enhances the chance of pyrene molecules in excited states to collide with oxygen molecules and renders oxygen-sensitive fluorescence. Particularly, pyrene molecules can form excimer at higher molar concentrations, which give rise to longer-wavelength emission (at ~480 nm) than that of pyrene monomer (370–430 nm), that is, large Stokes shift. The above merits make pyrene and its derivatives very attractive oxygen probes. Pyrene and/or pyrene derivatives have been reported to detect oxygen after being incorporated into polymer matrix [10–12] or dissolved in solution [13]. It is noticed that, however, high concentration of pyrene molecules usually is needed in those oxygen sensors, with the aim of forming excimers. For example, 2 mM pyrene was adopted in toluene-based oxygen sensing [12, 13], and the concentration was further raised up to 10 mM in polymer-based sensors that the solid matrix precluded the free diffusion of probe dyes [11, 12].

With the recent progress of nanotechnology, novel pyrene-based oxygen sensors with high sensitivity have been developed, wherein pyrene derivatives are either chemisorbed onto nanoporous aluminium plate [14–16] or attached to quantum dots [17, 18]. Although the concentration of utilized probe dyes is greatly reduced, the synthesis of pyrene derivatives and related nanomaterials is not easy for nonchemists and is time-consuming. It is known that pyrene molecules can form excimers in micelles of nonionic detergents [19]. Such a self-assembly approach is commonly used to load dyes into biologically based nanocarriers [20, 21]. Different from solid polymers, the dissolved pyrene molecules can move freely inside micelles, and, on the other hand, oxygen molecules diffuse more efficiently to and fro the submicron-sized micelles. Those merits inspire us to adopt micelle to host pyrene molecules, so as to facilely construct a fluorescent oxygen sensor.

In this work, pyrene molecules are directly dissolved into hexadecyltrimethylammonium bromide (CTAB) micelle (pyrene@micelle) to build fluorescent oxygen sensors. The as-resultant pyrene@micelle sensors have nanosized dimension with dominant excimer emission. Their oxygen-sensitive fluorescence is investigated in terms of both intensity and lifetime, and respective calibration line is plotted. The merits of large Stokes shift, easy fabrication, and good oxygen sensitivity make the pyrene@micelle sensors very attractive for fluorescent oxygen sensing.

## 2. Material and Methods

### 2.1. Materials

Pyrene (98%) was procured from Sigma Aldrich. Cetyltrimethylammonium bromide (CTAB, 99%) and absolute ethyl alcohol (99.7%) were procured from Beijing Lanyi Chemical Corporation (Beijing, China). All reagents were commercially available and used as received without further purification. High-purity deionized water (18.25 MΩ·cm) was produced using Aquapro EDI2-3002-U ultrapurified water system (http://www.aicwater.com.cn/).

### 2.2. Preparation of Pyrene@Micelle Oxygen Sensor

To 3 mL CTAB water solution (20 mM), different amounts of pyrene ethanol solution (5000 ppm) were added to result in pyrene@micelle with a concentration of 0.02, 0.06, 0.2, 0.4, 0.6, and 0.8 mM, respectively. The mixing took place under sonication for 20 minutes at 25°C and then was left still for 2 hours before further characterization including hydrodynamic size measurement and oxygen sensitivity test.

### 2.3. Characterization

Hydrodynamic size of pyrene@micelle aqueous dispersion was determined by dynamic light scattering (DSL), using a Zetasizer Nano instrument (Malvern Instruments, Malvern, UK). Steady-state fluorescence spectra were recorded on a LS55 fluorescence spectrophotometer (PerkinElmer). The fluorescence decay curves were measured by a time-correlated single-photon counting system (TCSPC), Fluorocube-01-NL (HORIBA Scientific), and the sample was excited by a pulsed ultraviolet light-emitting diode (373 nm, Nichia NSHU590E). The sample was placed in a 1 cm quartz cuvette and all the characterizations were performed at 25°C.

### 2.4. Pyrene@Micelle Calibration and Experimental Setup

The calibration was carried out in a cuvette filled with 2 mL of pyrene@micelle aqueous dispersion (0.8 mM), which was put inside a sealed plastic bag (vol. = 80 L) with an inlet and outlet. The plastic bag was vacuumed firstly before each operation and then inflated with an O_2_ (99.6%)/N_2_ (99.6%) gas mixture with various ratios, provided by a WITT gas mixer (type KM60-2, http://www.wittgas.com/, Germany), in the range of 1–25% with an accuracy of 1% absolute. In addition, 50% O_2_/N_2_ mixture, pure O_2_ and N_2_ were provided. Different dissolved oxygen (DO) concentrations were obtained by aerating the sample solution with the gas mixture for 3 h. All spectra measurements were performed at 25°C. The DO concentrations in pyrene@micelle dispersion were deduced according to that in oxygen-saturated solutions (43 ppm) based on the solubility equation of oxygen in water.

## 3. Results and Discussion

### 3.1. Synthesis and Characterization of Pyrene@Micelle Sensors

Initially, the CTAB micelle solution was turbid with the addition of pyrene and then became transparent again after being ultrasonicated for 20 minutes, with slightly opalescent color. Such a solubility transition indicates that pyrene molecules are completely dissolved in CTAB micelles; that is, pyrene@micelle is formed. It is important to note that the pyrene@micelle sensors are very robust as they can be recovered from turbid state after longtime storage simply by ultrasonication.

**Figure 1:**
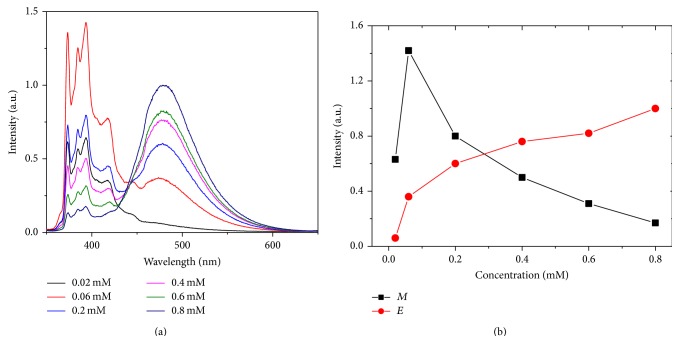
(a) Emission spectra (*λ*_ex_ = 337 nm) of pyrene@micelle solution with different concentrations of pyrene (0.02–0.8 mM). (b) Concentration-dependent fluorescent intensity of pyrene monomer (*λ*_em_ = 393 nm, the highest peak in monomer emission labeled as *M*) and excimer (*λ*_em_ = 474 nm, labeled as *E*). Data are calculated from (a).

In order to construct pyrene@micelle oxygen sensors, different amounts of pyrene were dissolved into CTAB micelles in order to render dominant excimer emission.  Figure 1(a) shows emission spectra of the resultant pyrene@micelle at different concentrations. The well-defined emission at 370–430 nm is attributed to pyrene monomer, and the broad band emission peaked at ~474 nm to pyrene excimer. Basically, with the increase of pyrene concentration, the excimer emission of pyrene@micelle is gradually enhanced at the expense of monomer emission. But it needs to point out that the monomer emission increases concomitantly with the excimer at low concentrations (e.g., 0.02–0.06 mM). The reason may be that the increased quantity of pyrene molecules is much bigger than that consumed through formation of excimer. Such a transition from monomer to excimer in pyrene@micelle can be clearly observed by the concentration-dependent fluorescence, as displayed in Figure 1(b). It can be seen from the figure that in pyrene@micelle the excimer emission is dominant over monomer when the concentration is higher than 0.3 mM. With the consideration that too much ethanol introduced by pyrene solution may influence the stability of CTAB micelle, 0.8 mM pyrene was adopted to build pyrene@micelle sensor in this work.

**Figure 2:**
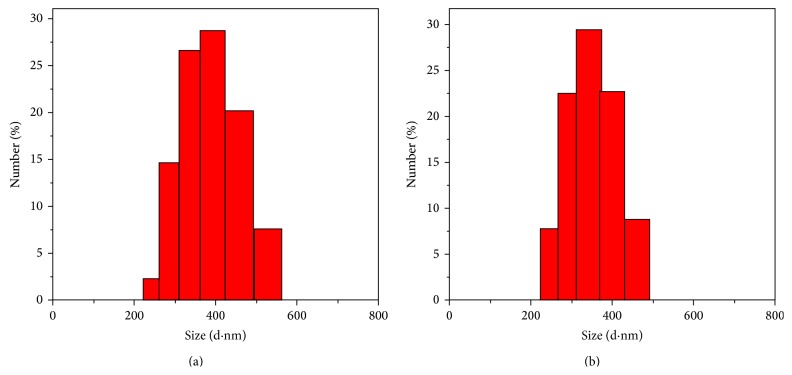
Hydrodynamic size of the CTAB micelles before (a) and after (b) addition of pyrene solution (measured at 25°C).

Hydrodynamic size of CTAB micelles before and after addition of pyrene is characterized by a Zetasizer analyzer, as shown in Figure 2. The size is determined to be around 386 nm and 348 nm, respectively. Apparently, the size of CTAB micelles is decreased, instead of increasing, after encapsulation of pyrene. This abnormal phenomenon is attributed to the influence of introduced ethanol, which increases the CMC in mixed solution [22].

### 3.2. Oxygen Sensitivity and Calibration of Pyrene@Micelle Sensors

**Figure 3:**
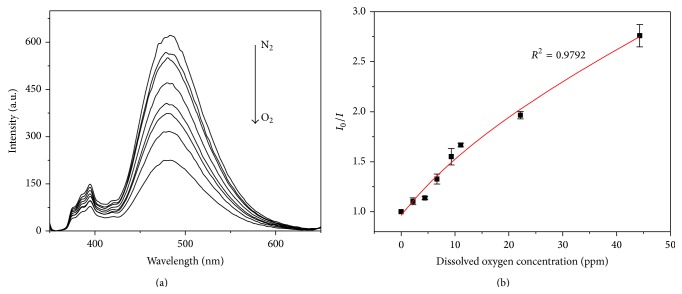
Oxygen sensitivity of pyrene@micelle solution (0.8 mM): (a) emission spectra at 337 nm excitation at various oxygen concentrations; (b) Stern-Volmer plot of fluorescence intensity of pyrene excimer. The experiment data were calculated from (a).

Pyrene@micelle solution was equilibrated with a gas mixture with various O_2_/N_2_ ratios, and their oxygen sensitivity was firstly tested on the basis of steady-state fluorescence.  Figure 3(a) shows the response of the emission spectra towards dissolved oxygen (DO) under 337 nm excitation. From the top to the bottom lines, the DO concentrations are 0.0 (nitrogen saturated), 2.21, 4.43, 6.64, 9.3, 11.07, 22.15, and 44.3 ppm (oxygen-saturated) in sequence. It can be seen clearly that the monomer and excimer emission of pyrene@micelle are both sensitive to oxygen. By defining *R* as the emission intensity, the sensitivity of the sensor can be expressed by the overall quenching response to dissolved oxygen [23],

(1) $$\begin{array}{c} Q=\frac{\left(R_{{\text{N}}_{2}}-R_{{\text{O}}_{2}}\right)}{R_{{\text{N}}_{2}}}, \end{array}$$

where *R*_N_2__ and *R*_O_2__ represent the emission intensity of the sensor in fully deoxygenated and fully oxygenated solutions, respectively. The as-obtained value of *Q* of monomer and excimer is around 47% and 65%, respectively. Apparently, the excimer emission is more sensitive to oxygen than monomer emission. It is necessary to note that oxygen sensitivity of pyrene excimer in micelles is lower than that in silicon polymer (*Q* = 92.5%) [12]. This reduced sensitivity might be due to the predissolved gas in CTAB micelles which weakens the fluorescence quenching by oxygen.

**Scheme 1:**
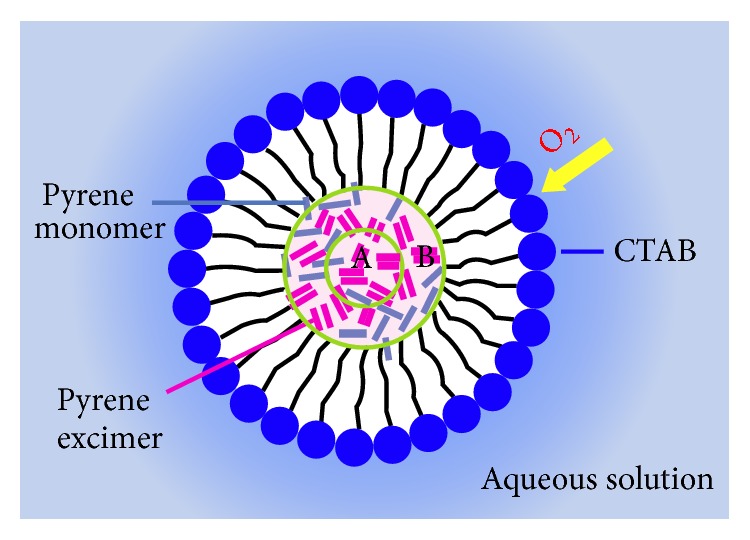
Cross-section of pyrene@micelle sensor (not to scale). The encapsulated pyrene monomers (labeled as blue bars) are free to form into excimers (pink bars), which can be approximately grouped as site A or site B according to their accessibility to oxygen.

In previously reported pyrene- (or pyrene derivatives-) based oxygen sensors, the oxygen-quenching process can be described by either linear [12, 17, 18] or nonlinear Stern-Volmer equation [16], corresponding to homogeneous and heterogeneous microenvironments of probe dye, respectively.  Figure 3(b) depicts the Stern-Volmer plot between the excimer emission intensity at 474 nm and DO concentration. It is observed that the plot follows a nonlinear behavior, which can be fitted by the following Stern-Volmer equation based on a two-site model (with a correlation coefficient of >0.979) [24–27]:

(2) $$\begin{array}{c} \frac{\tau_{0}}{\tau }=\frac{I_{0}}{I}={\left[\;\frac{f_{1}}{1+K_{\text{S}{\text{V}}_{1}}\left[{\text{O}}_{2}\right]}+\frac{f_{2}}{1+K_{\text{S}{\text{V}}_{2}}\left[{\text{O}}_{2}\right]}\;\right]}^{-1}, \end{array}$$

where *I*_0_ and *τ*_0_ are fluorescence intensity and lifetime in the absence of oxygen, *I* and *τ* are the fluorescent intensity and lifetime at a given DO concentration [O_2_], *f*_1_ and *f*_2_ are the emission fraction of the probes in different environment, and *K*_SV_ is the Stern-Volmer quenching constant of the different components. The line is the fitting function described by (2) and yields *K*_SV_1__ (*f*_1_ = 0.33) and *K*_SV_2__ (*f*_2_ = 0.67), 0.01 and 0.1, respectively. Since oxygen molecules are inwards diffused into pyrene@micelle from aqueous solution, the emissive pyrene excimers that are close to hydrophobic tail of CTAB (denoted as site B) are prone to be quenched in comparison to those in the core (site A), as illustrated in Scheme 1.

**Figure 4:**
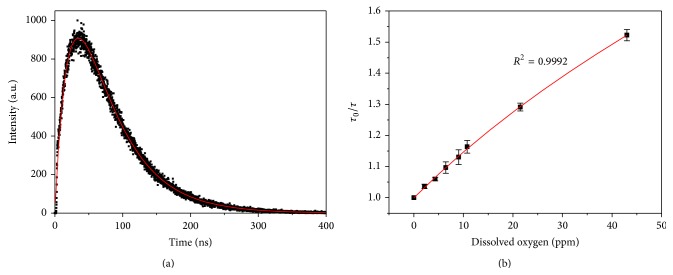
(a) A representative fluorescence decay curve of pyrene@micelle sensors monitored at 474 nm (*λ*_ex_ = 373 nm, at 25°C). The red line is the fitting function of (3). (b) Stern-Volmer plot of lifetime of excimer in pyrene@micelle sensors. The experiment data were acquired by fitting oxygen-sensitive decay curves.

Fluorescence intensity-based measurements usually suffer from variation of the sensor concentration and drifts of the optoelectronic system, such as lamps and detectors. In contrast, lifetime-based techniques can overcome these drawbacks and are more reliable. The excimer fluorescence decay curves of pyrene@micelle at different concentrations of DO are then measured, and a representative one is displayed in Figure 4(a). The growth part reflects the transition of pyrene molecules from excited monomer to excimer [18]. Since most of the pyrene monomers within micelles are converted into excimers under this concentration (demonstrated by the dominant excimer emission in Figure 1), the growth component is coincident with the decay kinetics of the monomer. By fitting the decay curve with a two-exponential equation,

(3) $$\begin{array}{c} I=I_{0}\left[\;\exp\left(\;-\frac{t}{\tau_{1}}\;\right)-\exp\left(\;-\frac{t}{\tau_{2}}\;\right)\;\right]. \end{array}$$

Lifetime of pyrene excimer can be determined, that is, *τ*_2_, along with that of monomer, *τ*_1_.  Figure 4(b) depicts the Stern-Volmer plot of the excimer lifetime of pyrene@micelle versus DO concentration. It can be well fitted by the nonlinear Stern-Volmer equation (2) and gives rise to *K*_SV_1__ (*f*_1_ = 0.41) and *K*_SV_2__ (*f*_2_ = 0.59), 0.002 and 0.02, respectively. With the consideration of quenching constants obtained from fluorescence intensity-based measurements, it can be concluded that (i) fluorescence lifetime does provide more accurate calibrating than intensity does, as revealed by the high correlation coefficient of >0.999; (ii) excimers in site B are distributed within a thin shell of the whole dissolved pyrenes because their proportions (*f*_1_ and *f*_2_) are comparable; (iii) the quenching constant of excimers in site B is ten times of that in site A, for both fluorescence intensity- and lifetime-based measurements, suggesting that reducing the diameter of micelle-typed sensors may greatly improve their oxygen sensitivity.

## 4. Conclusions

In summary, a micelle-typed fluorescent oxygen sensor was facilely constructed by dissolving pyrene molecules into CTAB aqueous solution. The pyrene@micelle sensors were of nanosized dimension, and low concentration of pyrene (e.g., >0.8 mM) was sufficient to render dominant excimer emission. The excimer fluorescence is sensitive to dissolved oxygen with a quenching response of 65%. Both the fluorescence intensity- and lifetime-based oxygen sensitivities were well fitted by a nonlinear Stern-Volmer equation. Based on the fitting parameters, microenvironments of pyrene excimers inside micelles were then classified as two types: one is close to CTAB molecules and easy to access by oxygen; the other is in the core of micelle and difficult to be accessed by oxygen. Therefore, it is expected that the sensitivity of micelle-typed sensors can be further improved if their size is reduced, and related work is being carried out. Considering the simplicity, sensitivity, and robustness of the pyrene@micelle sensors, they may find applications in industrial or environmental fields.
